# Effect Size of Testimonials on Treatment Choice in PTSD

**DOI:** 10.3389/fpsyt.2013.00018

**Published:** 2013-03-25

**Authors:** Brandon M. Smith, L. Lee Glenn

**Affiliations:** ^1^College of Nursing, East Tennessee State UniversityJohnson City, TN, USA; ^2^Institute for Quantitative Biology, East Tennessee State UniversityJohnson City, TN, USA

The recent study by Pruitt et al. (2012) that was published in *Behavior Research and Therapy* concluded that “For a critical unreached treatment sample, those who are (PTSD) treatment naïve, positive patient testimonials offer a mechanism in which to make effective treatments more appealing and accessible.” However, that conclusion is not supported by the evidence of their study because (1) the effect sizes of their study are all very small, and (2) after correcting for the number of hypothesis tests using the Bonferroni method, none of their results are statistically significant, as explained below.

First, although the study claims that video testimonials positively affect the perception of available PTSD interventions for treatment naïve individuals, the average standardized effect sizes (as expressed by the Pearson correlation coefficient) for each group were far too low to warrant these claims. The averages for the community and undergraduate populations tested in the study were *r* = 5.8 and 4.8%, respectively, which indicate only a minor difference from zero. For those participants who have received prior psycho- or pharmaco-therapy, the average effect size was even lower, at 3.3 and 1.9%. Overall, the effect of the testimonials for both groups combined was 5.3%, which is neither clinically significant nor statistically significant (*p* = 0.179). Therefore, the conclusion from the study that positive video testimonials on available PTSD treatments affect the opinion of treatment naïve individuals is not supported by the data in this study.

Second, there were 40 statistical tests in the results section of the above study related to testimonials. Two out of the 40 would be expected to be significant by chance alone due to Type II error. That is, no correction was made for the number of tests conducted. After applying the Bonferroni correction to the data in the study, the alpha level was 0.00125 and none of the testimonial related findings were statistically significant. This indicates that the responses from the video testimonials were likely from chance associations, not meaningful responses to the study. Therefore, the conclusion that the video testimonials positively affect views on treatment is contradicted by the lack of statistical significance for any of the tested groups in the study.

Many strengths are found in the study, including an important topic, excellent sample size, high measurement reliability and validity, a clear description of methods and results, and a comprehensive discussion section, demonstrating both a passion and dedication to the study of PTSD. Despite these strengths, the effect sizes for the video testimonials were both clinically and statistically insignificant. Therefore, the study conclusion that video testimonials positively affect perceptions of treatment, is not sufficiently supported at this time to warrant use with the PTSD population.
